# GPR-Based Water Leak Models in Water Distribution Systems

**DOI:** 10.3390/s131215912

**Published:** 2013-11-25

**Authors:** David Ayala-Cabrera, Manuel Herrera, Joaquín Izquierdo, Silvia J. Ocaña-Levario, Rafael Pérez-García

**Affiliations:** 1 FluIng-IMM, Universitat Politècnica de València, Camino de Vera s/n Edif. 5C 46022, Valencia, Spain; E-Mails: daaycab@upv.es (D.A.-C); silocle@upv.es (SJ.O.-L.); rperez@upv.es (R.P.-G.); 2 BaTir, Université libre de Bruxelles, Av. F. Roosvelt 50 B-1050, Brussels, Belgium; E-Mail: mherrera@ulb.ac.be

**Keywords:** water leak identification, ground penetrating radar (GPR), non-destructive methods, signal and image processing and analysis, water supply systems

## Abstract

This paper addresses the problem of leakage in water distribution systems through the use of ground penetrating radar (GPR) as a nondestructive method. Laboratory tests are performed to extract features of water leakage from the obtained GPR images. Moreover, a test in a real-world urban system under real conditions is performed. Feature extraction is performed by interpreting GPR images with the support of a pre-processing methodology based on an appropriate combination of statistical methods and multi-agent systems. The results of these tests are presented, interpreted, analyzed and discussed in this paper.

## 1. Introduction

Water is valuable, but challenging to manage. It has been calculated that many water distribution systems (WDSs) around the world lose more than 40 percent of the clean water pumped into the distribution system because of leaks before that water reaches end consumers [1]. By reducing the amount of water leaked, WDS managers can reduce the amount of money and energy wasted on producing and pumping water, increase system reliability and more easily satisfy present and future consumer needs. Having access to sufficient information regarding leaks is a complex task. Many water utilities struggle to measure and locate leaks in their distribution networks.

Improved leakage management in WDSs is one of the intelligent solutions that can make a difference. The use of different types of smart sensors to gather data and the application of advanced analytics could provide valuable information on the location of leaks in the network. Specifically, non-destructive methods, such as ground penetrating radar (GPR), can help locate primordial leaks and, so, help resolve the problem, while avoiding social and economic costs.

In [2], a review of the various pipeline inspection techniques most commonly used in WDSs systems and wastewater collection is performed. These techniques are divided into four groups: (a) visual techniques; (b) electromagnetic and radio frequency techniques; (c) acoustic and vibration techniques; and (d) other techniques. Closed-circuit television (CCTV) and sewer scanner and evaluation technology (SSET) are highlighted in the first group (visual techniques). The second group (electromagnetic and radio frequency techniques) consists of magnetic flux leakage (MFL), eddy current technique, hydroscope technology (HT), rapid magnetic permeability scan (RMPS), low frequency electromagnetic field (LFEM), passive magnetic fields (PMFs), time domain ultra wideband (UWB) and ground penetrating radar (GPR). The third group (acoustic and vibration techniques) includes sonar, vibro-acoustics, impact echo/spectral analysis of surface waves and correlator and listening sticks for leaks. The last group (other techniques) includes infrared thermography, continuous wave Doppler sensing technique, laser surveys, combined techniques (broadband electromagnetics/wave impedance probe (WI), pipe inspection real-time assessment technique (PIRAT) and the Sahara Project.

Among these techniques, the most popular for locating leaks in water supply systems are those included in the acoustic and vibration technique group, infrared thermography and GPR [3,4]. Acoustic methods detect the acoustic wave generated by the leak based on correlation analyses of the wave velocity of the sound emitted by the pipe being inspected. Such methods are widely used to identify leaks in fluid-filled metal pipes [5]. The main drawback of the aforementioned methods is their inefficiency in detecting leaks in non-metallic pipes (e.g., Polyvinyl chloride (PVC) pipes) [6]. Infrared methods detect thermal contrasts caused by the difference of temperature between ground and water. However, even though easy to implement, these methods produce errors when there are considerable differences in temperature. Furthermore, it is not possible to use these techniques in summer and winter, due to the absence of significant differences between ground and water [4]. GPR is shown as an effective nondestructive tool that favors inspection of WDS by demarcating on GPR image (radargrams) contrasts between the leaked water and the surrounding soil that are caused by differences in dielectric characteristics [7,8].

The use of GPR as a method for locating leaks in WDS has become more widespread in recent years. In this sense, there is fieldwork, such as [9], performed on urban pipe sections. Pre-processing of the obtained images is performed by using low-pass filters to identify leaking PVC pipes. Another representative fieldwork is reported in [10]. In this case, a plastic pipe (PVC) was drilled and buried in the ground; and, an analysis was made using raw images. Likewise, there is fieldwork using a combination of methods. Such is the case of [11], which combines GPR assays with electrical potential and geochemical assays to detect leaks in non-pressurized non-metallic pipes. In this case, leaks are identified from raw GPR images. Laboratory tests are also employed in finding leaks using GPR. Works, such as [12,13], concentrate on plastic pipes. In these cases, pre-processing includes background removal and image filtering, respectively. A combination of survey work conducted both in the field and in the laboratory is presented in [14]. In this paper, various tests on leaks in plastic and metallic pipes were performed. In this work, Kirchhoff migration and the Hilbert transform were used as pre-processing methods. These assays are promising with respect to the use of GPR in finding leaks in WDS. However, most of these assays are based only on the location and interpretation of hyperbolas generated either in raw or pre-processed images.

Identifying leaks by GPR images is not an easy task and requires a high level of expertise by the operator. Added complications include the complex spatial arrangement of many networks, along with the steady growth in the supply infrastructure of cities. These aspects greatly increase the difficulty in using and interpreting data obtained with GPR in detecting leaks and analyzing the results, thus reducing the potential for solving problems and increasing the need for highly qualified personnel. This paper attempts to address this issue with the extraction of features visually and numerically. To this purpose, laboratory tests are performed in which we seek to extract features of water leakage from GPR images. Feature extraction is performed by interpreting the GPR images with the support of a pre-processing methodology based on an appropriate combination of statistical methods and multi-agent systems. Subsequently, these features are observed in a field test on a real water leak-case in a WDS. The ultimate goal of these processes is to extract features to feed intelligent automatic processes for the (automatic) detection of leaks in WDS using GPR images. This research thus seeks to encourage the use of these tools in finding leaks by non-highly qualified personnel and, thus, promoting improved management of WDS.

The paper is organized as follows. In the first section, we have presented a brief introduction and cited relevant papers. The second section presents the characteristics of the tests performed. The third section presents an analysis of typical GPR images by locating and interpreting hyperbolas. The fourth section discusses the numerical contrast images of a non-leaking laboratory empty pipe and then a series of tests with water and leakage. The use of a race-agent algorithm as a pre-processing tool for GPR images and its application to the images of the laboratory tests are proposed in Section 5. The following section presents a contrast analysis, similar to that performed in Section 4, but using pre-processed images. 3D models of the interpretations obtained in Sections 4 and 5 are then presented in Section 7. The eighth section presents the identification of a leak in fieldwork. Finally, a section of conclusions closes the document.

## 2. Data Capturing: Design and Layout of the Laboratory Tests

**Figure 1.**
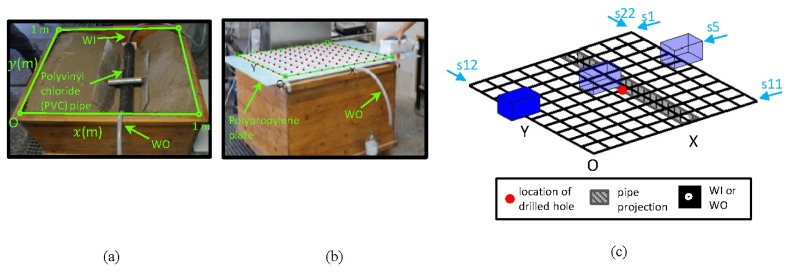
Laboratory layout. WI, water input; WO, water output.

This section presents the layout of the laboratory tests. In this set of tests, a pipe commonly used in WDSs is buried in dry soil in a tank (see Figure 1a). The characteristics of the buried pipe are: (a) PVC; (b) diameter of 100 mm; (c) length of 0.95 m; (d) hole drilled for simulating the leak in the central part of the pipe; (e) two points for water input (herein termed WI) and output (herein termed WO) with connections at the ends. A wooden tank measuring 1.0 m × 1.0 m × 0.70 m was used. After the pipe was positioned, its supports were removed, and it was then covered with dry soil. The surface of the tank was covered with a polypropylene plate. Eleven paths parallel to the *x*-axis and another eleven paths parallel to the *y*-axis were marked on this plate. These 22 paths were spaced 0.10 m apart (see Figure 1b) and, so, formed the sampling grid (see Figure 1c). Onto each line of this mesh, the GPR antenna was slipped. The image produced by sliding the antenna is termed a profile in this document (see, for example, s5 in Figure 1c). Additionally, we distinguish between horizontal and vertical profiles when referring to the profiles parallel to the *x*- and the *y*-axes, respectively.

The GPR equipment used in each survey corresponds to a commercial monostatic antenna with a central frequency of 1.5 GHz. The parameters of the equipment correspond to 120 traces/s, 512 samples/trace and 20 ns/512 samples. Two tests with the aforementioned features of 22 profiles were performed. These two tests are differentiated one from the other in that the first was without water inside the pipe (nor leaked water), while the second test had water inside the pipe and leaked water. These two situations are called initial and final state, respectively, in this paper.

## 3. Analysis of the Location and Identification of Hyperbolas from the Raw Images

**Figure 2.**
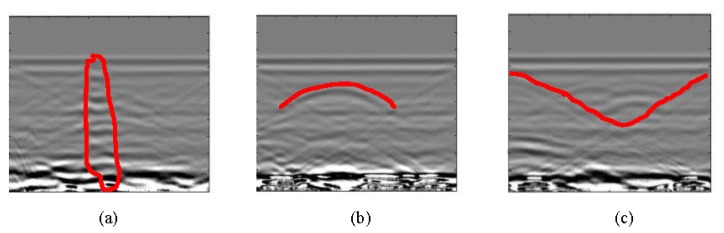
Identifiable forms in raw images in Sections 3.1 and 3.2: (a) distorting vertical strip; (**b**) hyperbola; (**c**) triangles.

This section presents a typical analysis to identify anomalies (leaks in this case) in the raw images. This analysis consists in locating and identifying hyperbolae in the GPR images, according to the scheme presented in the previous section. These tests are performed on the horizontal and vertical profiles for both the initial and end states. To facilitate the interpretation of raw images in Sections 3.1 and 3.2, various examples of target forms are sketched in Figure 2. Incidentally, Figure 2a corresponds to Figure 5a, Figure 2b to Figure 5c and Figure 2c to Figure 5g.

### 3.1. Initial State: Raw Images

**Figure 3.**
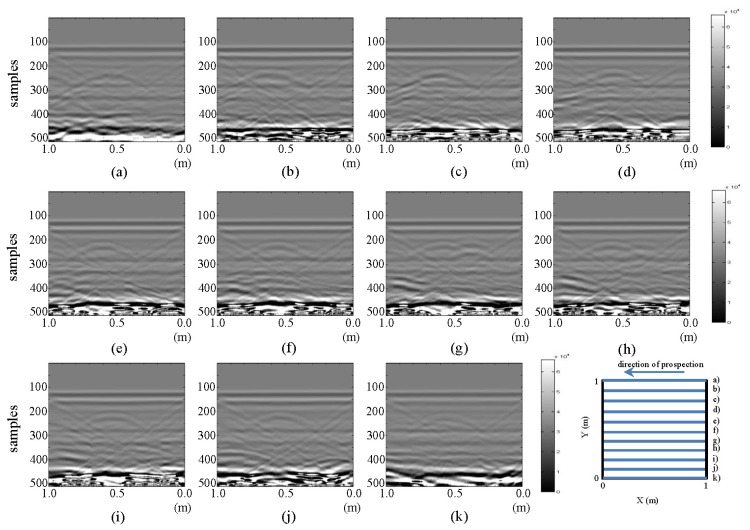
Horizontal profiles: raw images for the initial state. (**a–k**) are profiles s1 to s11, respectively.

**Figure 4.**
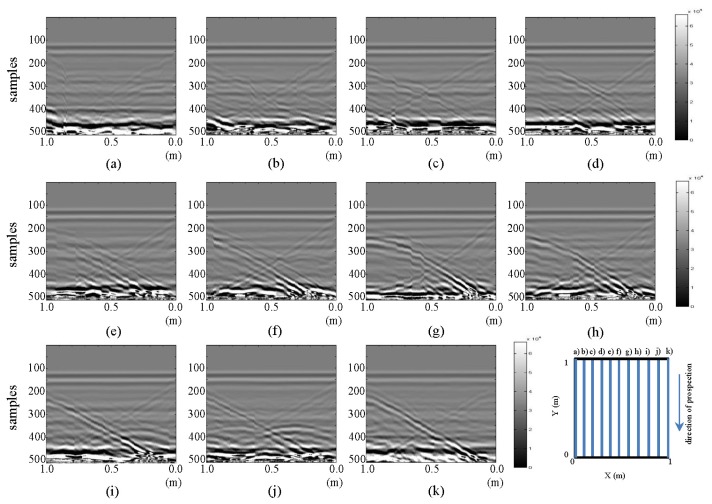
Vertical profiles: raw images for the initial state. (**a–k**) are profiles s12 to s22, respectively.

The resulting images from the horizontal and vertical profiles of the tests performed in the laboratory for the initial state are presented in Figures 3 and 4, respectively.

All the images presented in Figure 3 exhibit a hyperbola, whose center is located at approximately 0.6 m in all cases. This location coincides with the location of the PVC buried pipe. In this same figure, in parts (f), (g) and (h), a new anomaly, a new hyperbola located to the right of the hyperbola associated with the buried pipe, can be identified. The new hyperbola increases in intensity in Figure 3g. In this case, it is known that this anomaly cannot represent the leak, since we are still analyzing the initial state. However, in an uncontrolled case, this feature could lead to confusion.

All the images depicted in Figure 4 show how the hyperbola representing the pipe in Figure 3 no longer appears. This is consistent with the schematic configuration proposed for the test, given that vertical profiles are being considered. However, formations (triangles) inclined from the tank walls, which were also seen in the horizontal profiles (Figure 3), can be observed. In all the images in Figure 4, a gradually spanning structure can be observed: for parts from (a) to (d) between samples 275–325; for part (e) between samples 250–325; for (f) to (h) between samples 225–275; for part (i) between samples 275–325; and for (j) between samples 300–325. This pattern corresponds to the signal response for the now longitudinally placed pipe.

### 3.2. Final State: Raw Images

**Figure 5.**
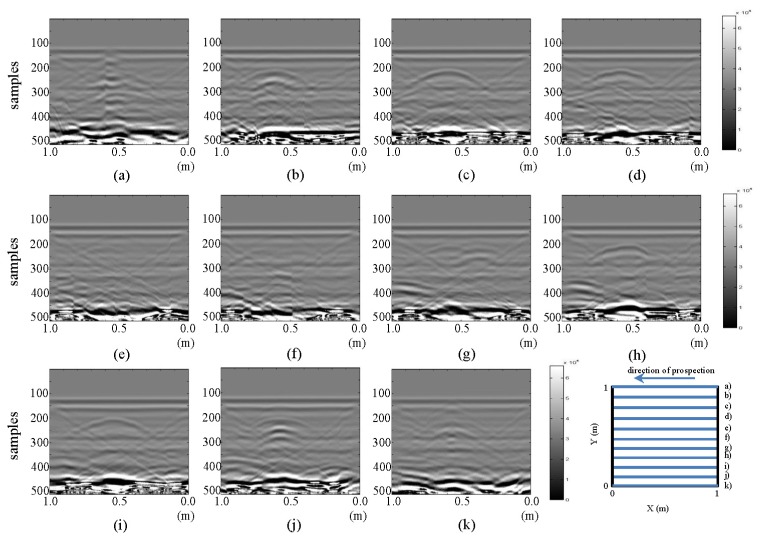
Horizontal profiles: raw images for final state: (**a–k**) are profiles s1 to s11, respectively.

**Figure 6.**
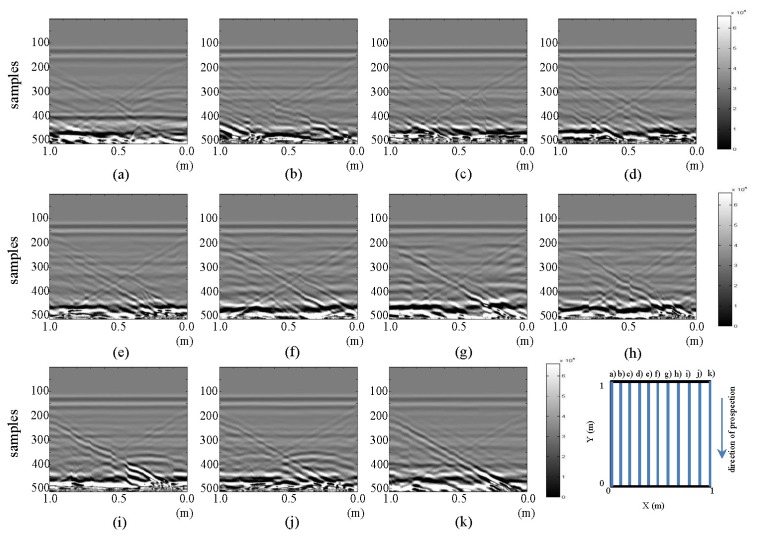
Vertical profiles: raw images for final state: (**a–k**) are profiles s12 to s22, respectively.

The resulting images of horizontal and vertical profiles of the tests performed in the laboratory for the final stage are presented in Figures 5 and 6, respectively.

In Figure 5, in images (a) and (k), a vertical strip that distorts both images can be seen between 0.5 and 0.6 m. This effect is attributed to the presence of water in WI and WO. This effect, which is more accentuated in (a), is more clearly seen when contrasting these images with their corresponding images in Figure 3: the area containing the deformed strip in Figure 5 contained (in Figure 3) the shape of the hyperbola previously identifying the buried pipe. In images (b) and (j) of Figure 5, a new hyperbola surrounded by another upper hyperbola (pipe initial hyperbola) can be identified. By observing profiles from (b) to (j), we can say that the hyperbola we had considered to represent the pipe decreases in intensity as the position of the profile nears the point of the leak. In effect, the hyperbola is almost invisible in images (e) and (f). We can also observe how in image (f), two hyperbolas are identified (very faintly), where, initially, only one hyperbola appeared. In (g), this second hyperbola as obtained in the initial state (close to the hyperbola of the pipe) is observed. If we had not previously had the image without water, this second hyperbola would surely have been interpreted as a leak. This shows the need to extract patterns for interpretation and leak detection in water supply systems and, so, avoid common mistakes in field interpretation. This additional hyperbola may result from any object (such as stones) near the pipeline.

Images in Figure 6, in contrast, show similar characteristics to the respective images of the initial state. However, in (f) and (g), a contraction of the area we had identified as the pipe in Figure 4 can be observed between 0.5 and 0.4 m (distance axis) and samples 225 and 275 (depth axis). This contraction is better observed in image g), which corresponds to the vertical section closer to the pipe. Furthermore, the position of the contraction is consistent with the location of the leakage point in the test.

After these various analyses of the different horizontal and vertical profiles corresponding to the leakage, we observe that identifying leaks directly from raw images is difficult. In fact, even considering the *a priori* information about the location of the leak, it is a challenge to distinguish all the above-mentioned features.

## 4. Analysis: Contrast between Raw Images for the Initial and Final States

**Figure 7.**
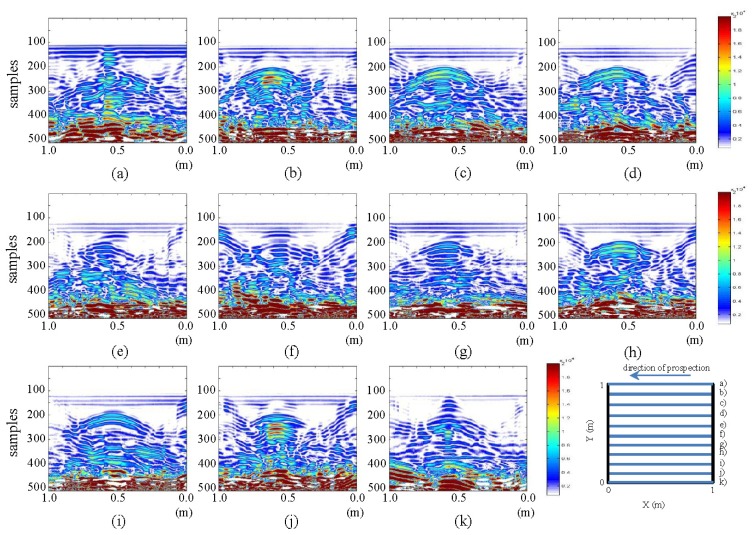
Horizontal profiles: contrasting raw images for initial and final states. (**a–k**) are profiles s1 to s11, respectively.

**Figure 8.**
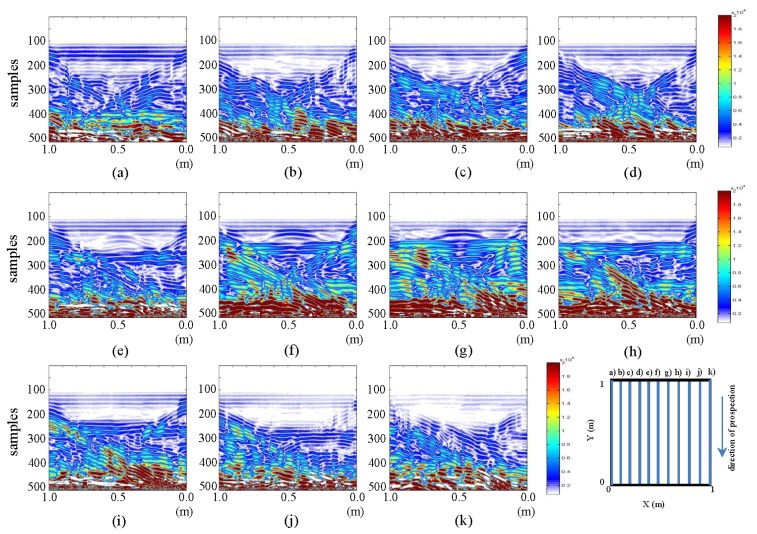
Vertical profiles: contrasting raw images for initial and final states. (**a–k**) are profiles s12 to s22, respectively.

In this section, since a suitable metric is available, we present a comparison between the initial and the final state of the tests performed for each raw image. Figures 7 and 8 show the differences in horizontal and vertical raw images, respectively.

The images in Figure 7 show the presence of water, which is the differentiating factor between the images of the initial and the final state. In parts (a) and (k) of the figure, the water in WI and WO can be easily seen. The water contained in the pipe can be observed in pictures from (b) to (j). The brightest part now corresponds to the water that has replaced the air contained in the pipe in the initial state. This shows that the difference in intensity identifies the specific fluid (air or water, in our case) contained in the pipe. Similarly, it is observed that the color intensity decreases as the location of the images nears profile (f) (the profile closest to the leak). Moreover, in images (e) to (g), between 0.4 and 0.6 m and between samples 150 and 200, a hyperbola that was not readily detectable in the respective raw images (and was missed in the interpretations) is now easily observed. This hyperbola is defined, according to the observation made *in situ*, by the rise in water leaked by capillarity. For images (e) and (f), the water contained in the pipe, on the right side (of the image), is demarcated with correspondingly lower intensity. This would indicate that the leak is running from left to right. This is because in that area, there is a mixture of soil and water, and this causes lower color intensity. A decrease in color intensity in the demarcated area of the pipe when approaching the leakage point (profile f) can also be observed. Additionally, some deformation is also observed below all the areas demarcated by the contrast, which is the result of the expected convolution (bearing in mind that the measurements are performed at depth).

In Figure 8, image (g) (which is the closest to the pipe and to the leakage point), an increase in the color intensity in the strip running almost through the entire profile can be observed. This strip, between samples 200 and 250, shows the addition of water in the pipe. In this same image and for the same strip, the effect described regarding the same image for the final state can also be seen. This is the effect of the contraction of the pipe at the point where the leak is located. In addition, there is a hyperbola at the top (a feature not seen in its respective final state image). Similarly, in (h) (the profile closest to the pipe and on the path where the pipe is located), the strip has lower color intensity than in (g), while being higher than in the closer profiles. Observe that this strip fades and diminishes in height, in the direction from (i) to (j) and from (f) to (e).

Likewise, a hyperbola in (e), (f) and (g), which gains color intensity as the point of leakage is approached (image g)) can be seen. From (f) to (e), the hyperbola loses strength and increases in size as a result of the increasing distance from the point of the leak.

## 5. Analysis of Location and Identification of Anomalies in Pre-Processed Images

In this section, we present the principles of the pre-processing algorithm used in this work. Subsequently, this algorithm is applied to the images obtained in the laboratory for the initial and final state. The results will be compared and analyzed later.

### 5.1. Multi-Agent System for the Pre-Processing Algorithm

**Figure 9.**
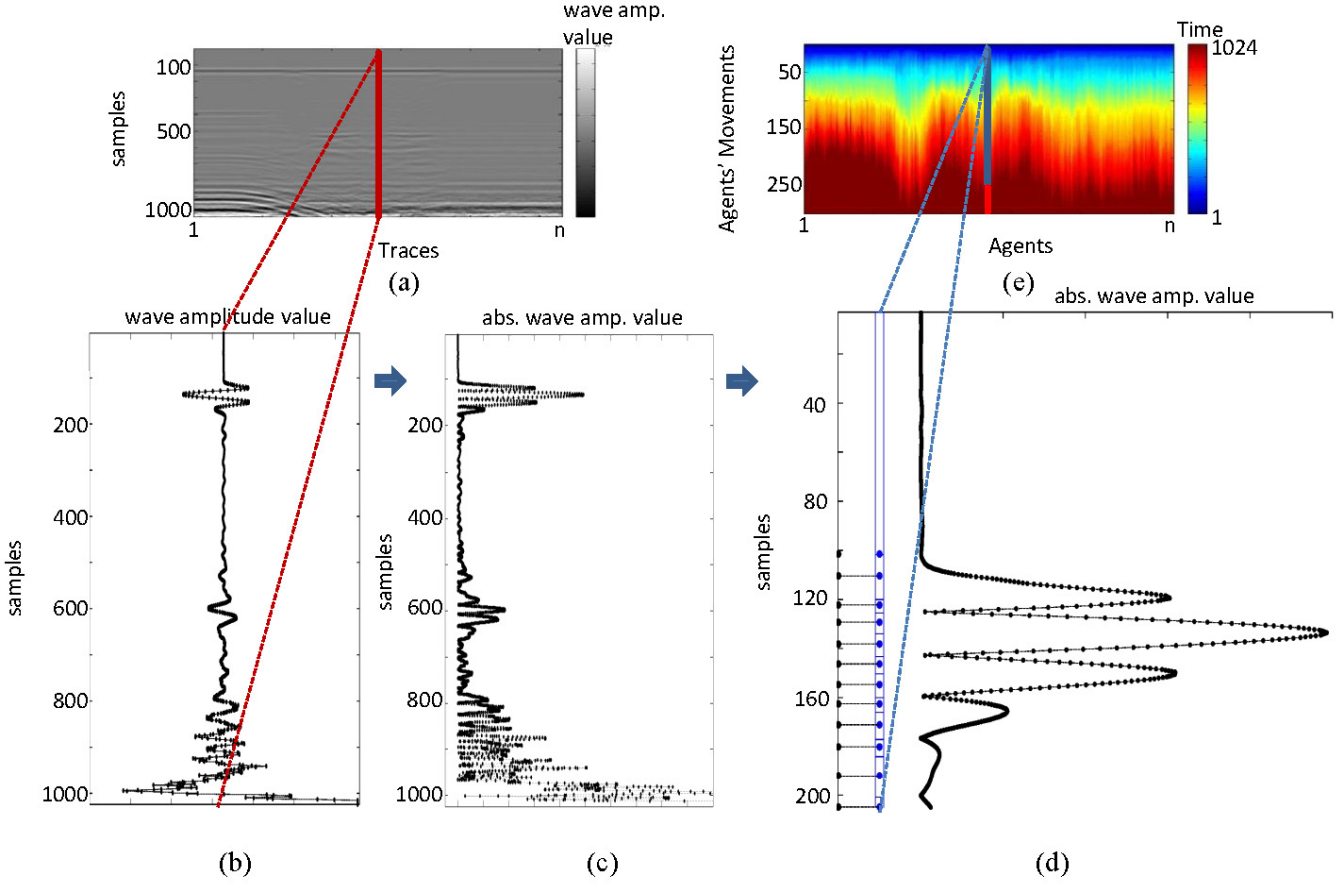
Principles of the ground penetrating radar (GPR) image pre-processing.

The analysis carried out in Section 3 is based on intensity differences demarcated by the wave amplitude generated in the images after the passage of the signal through various subsurface strata. The analysis we present in this section is based on time characteristics. In this analysis, the peaks (both maxima and minima) of the waves generated are extracted. The trend of the path of each trace and the average value of peak-to-peak time are studied. The basic principle assumes that the field is homogeneous, and thus, there should be a clear correspondence between the various peaks obtained. However, it should be noted that although the material is homogeneous, in practice, the measured values are different, even though very close together. Consequently, very different values demarcate anomalies in the image. These peaks were first extracted and numbered according to their occurrence in the trace. They were then placed in their respective positions in distance, and finally, the last value is used to fill an array of a certain size (512 in this work) (see Figure 9).

To quickly obtain this matrix, the algorithm proposed by [15] was used. In this algorithm, we obtain the above-mentioned values by means of a system based on multiple entities (agents) that search peaks by simulating a race. A multi-agent system consists of a population of autonomous entities (agents) situated in a shared structured framework (environment). The system is based on such tools as game theory (e.g., setting the agent preferences by means of a utility function), economics, biology, as well as artificial intelligence algorithms [16]. Agents operate independently, but are also able to interact with the environment and coordinate with other agents. This coordination may imply cooperation if the agent society works towards common objectives. Thus, in a cooperative community, agents usually have individual capabilities, which, when combined, will lead to solving the entire problem. However, cooperation is not always possible, and there are instances where agents are competitive and have divergent goals. In this later case, the agent should also take into account the actions of others [17]. However, even if agents are able to act and achieve their goals by themselves, it may be beneficial to partially cooperate and form coalitions for a better performance. When coordinating activities, either in a cooperative or a competitive environment, negotiation may prove a suitable method for solving conflicts among agents. Negotiation may be seen as the process of identifying interactions based on communication and reasoning with regard to the state and intentions of other agents [18].

**Figure 10.**
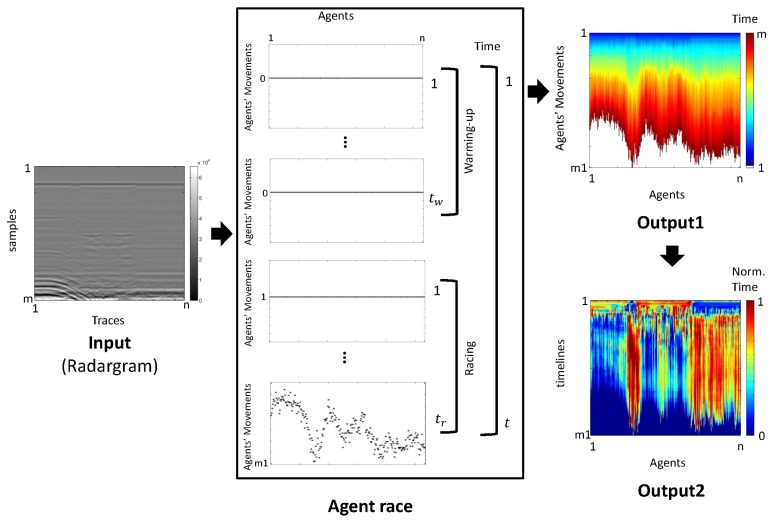
Scheme for the agent-race algorithm.

The pre-processing of GPR images used in this document was proposed in [15] and was termed an agent race. The algorithm has been developed in MatLab, is based on game theory and uses the multi-agent paradigm [16]. Agent racing provides an interpretation and a grouping method for data from GPR radargrams. In this pre-process, we reduce the amount of data in the initial radargram, while preserving its initial properties and the most relevant data, so that its ability to identify buried objects through suitable visualizations is preserved. The multi-agent approach makes analysis much quicker. The input to this algorithm is the resulting radargram of the GPR prospection, which consists of an *m*×*n*-sized matrix. The *n*-traces, of length *m*, that are generated are used in this work as parallel tracks for the *n*-agents to run. The race is an endurance test for the agents with a prize consisting in advancing one position depending on the effort made. Efforts are based on wave amplitude values in each column of the matrix (radargram). The agent race is comprised of two phases: (a) warm-up; and (b) competition. The race lasts a total time of *t* = *t_w_* + *t_r_* = *m*, where *t_w_* is the warm-up time and *t_r_* the competition time. The movements of an agent in *t_r_* are conditioned by the changing trend of the wave amplitude of the trace the agent runs. The race ends when time *t* has elapsed. The winner is the agent who manages to obtain more movements (reward) during this time. The output (output 1) of this process is a matrix of the size *m* 1 *l* × *n*, where *ml* = the maximum number of movements. The columns of this matrix describe the movement of the agents in relation to the competition. In this paper, we restrict the output (output 2) to the matrix of the size *t_r_* × *n* that collects the movements performed by each agent during the competition (see Figure 10). In this work, the various movements developed by the agents are termed time lines.

**Figure 11.**
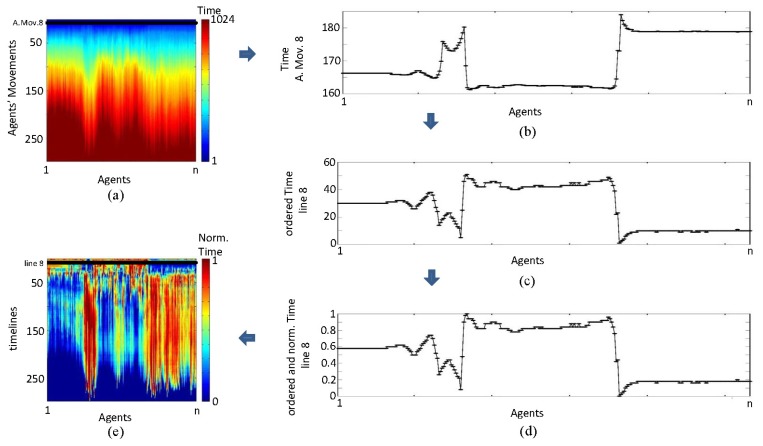
Output 2 building: (**a**) output 1; (**b**) time line; (**c**) ordered time line; (**d**) ordered and normalized time line; and (**e**) output 2.

On each time line, times obtained in the competition for each agent are ordered decreasingly. These time lines are then normalized. This produces output 2 (Figure 11), which is the matrix we use herein. This eliminates the delays caused by the gap between rows and enables more intense highlighting of anomalies that are very small and difficult to observe in raw images. However, care must be taken with this regularization, because although the anomalies are highlighted, their intensity is determined in relation to the ground prospected in each profile. This can cause visual errors in interpretation. Yet, even if this occurs, the interpretation based on the identification of various forms will enable zones of interest to be delimited, thus facilitating more complex analyses.

**Figure 12.**
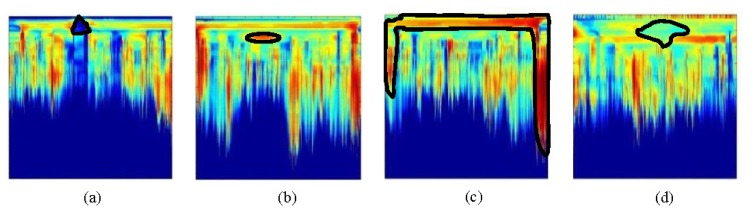
Identifiable target forms in pre-processed images in Section 5.2 and 5.3: (**a**) conical shape for water input (WI) and output (WO); (**b**) ellipse; (**c**) tank and measuring plate contours; and (**d**) water leak shape.

Again, to facilitate the interpretation of preprocessed images in Sections 5.2 and 5.3, examples of the more relevant forms are sketched in Figure 2. In this case, Figure 12a corresponds to Figure 15a, Figure 12b to Figure 15c, Figure 12c to Figure 15g and Figure 12d to Figure 16f.

### 5.2. Initial State: Pre-Processed Images

**Figure 13.**
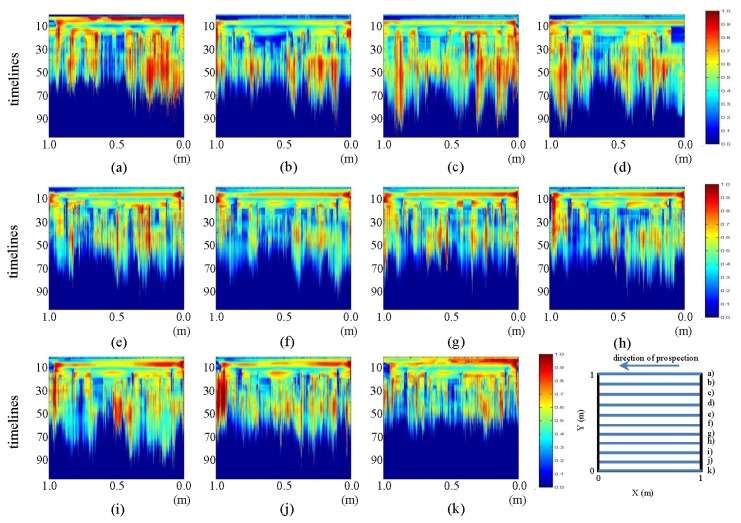
Horizontal profiles: pre-processed images for the initial state. (**a–k**) are profiles s1 to s11, respectively.

**Figure 14.**
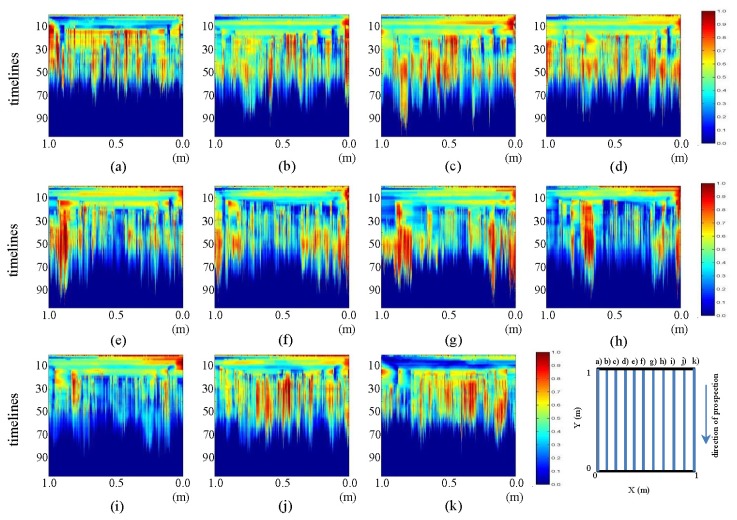
Vertical profiles: pre-processed images for the initial state. (**a–k**) are profiles s12 to s22, respectively.

The pre-processed images derived from the tests performed in the laboratory for the initial state show the horizontal and vertical profiles presented in Figures 13 and 14, respectively.

In Figure 13, an ellipse-shaped object is observed exactly in the place where the pipe was placed. In each image, it can be observed how the color intensity decreases from the center of the ellipse to its border. In images (a) and (k), an additional conical shape may be observed above this ellipse, which is not noticeable in the corresponding raw images. This image corresponds to WI and WO. Additionally, at about time line 10 in all the images (with variable intensity), the development of a horizontal figure can be observed that crosses all the images, which, at each of its two ends, joins an intense zone that demarcates a vertical figure. This horizontal formation is the polypropylene plate used for sliding the antenna during measurement, and the vertical forms correspond to the walls of the tank.

In the images in Figure 14, a certain relationship between the different color intensities of each profile can be observed. The same characteristic areas of the tank and the measuring plate, already observed in the pre-processed horizontal profiles, are demarcated with greater intensity. However, the inclination of this area, which was previously horizontal, can be observed from images (f) to (h). Moreover, in the central part, we note the non-appearance of the ellipse that appeared in the pictures above.

### 5.3. Final State: Pre-Processed Images

**Figure 15.**
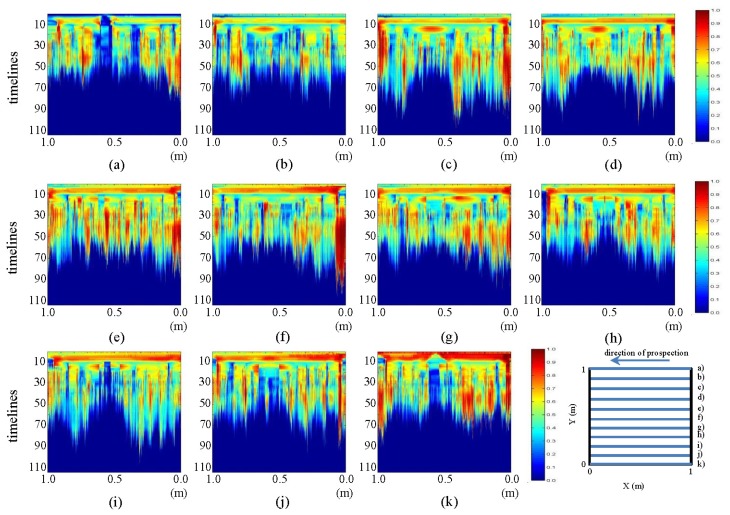
Horizontal profiles: pre-processed images for the final state, (**a–k**) are profiles s1 to s11, respectively.

**Figure 16.**
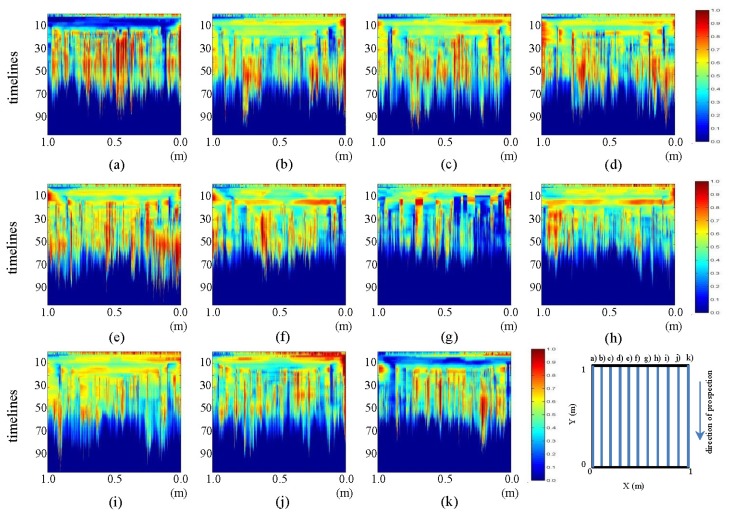
Vertical profiles: pre-processed images for the final state. (**a–k**) are profiles s12 to s22, respectively.

The pre-processed images derived from the tests performed in the laboratory for the final state, showing the horizontal and vertical profiles, are presented in Figures 15 and 16, respectively.

In Figure 15, it can be seen how the ellipse observed in the corresponding initial state image gains color intensity with the addition of water in the pipe for all the images. Likewise, in images (e) and (f), such intensity decreases. This feature was observed in the profile of the raw images and can be seen more clearly in the pre-processed images. Likewise, it can be observed in (a) and (k) that the intensity of the structure representing WI and WO increases with the addition of water to the system. It should be noted that this feature is clearly observed only after contrasting the initial and final states and not directly in the raw images. However, in the pre-processed image, this feature is easily identifiable. In images (g) to (k), the central ellipse loses intensity toward 0 m and fades in its central part. The contours generated by the tank and measuring plate are better delineated in these images.

In Figure 16, a discontinuity of color intensity in the form of an ellipse between images (e) to (g) can be observed. This discontinuity grows as it approaches profile (g) (location of the leak). It should be mentioned that this was not at all observable in the corresponding raw image. Now, in the pre-processed images, it becomes apparent.

## 6. Analysis: Contrast between Pre-Processed Images

**Figure 17.**
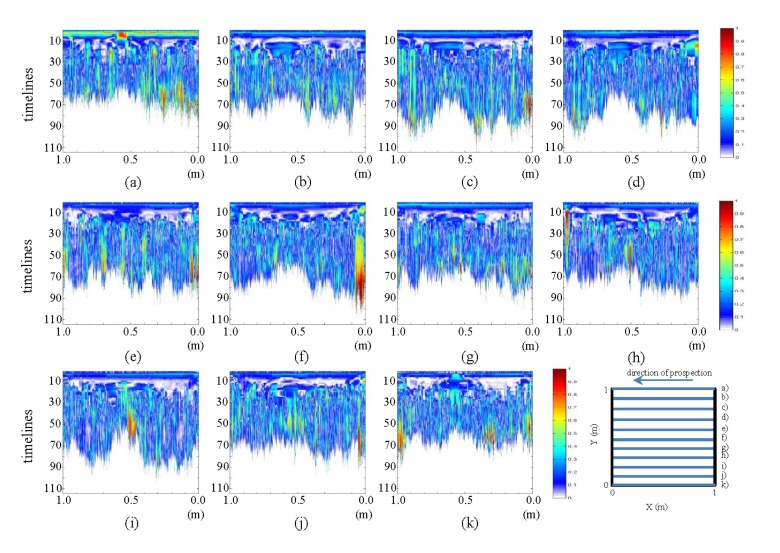
Horizontal profiles: contrast between pre-processed images. (**a–k**) are profiles s1 to s11, respectively.

**Figure 18.**
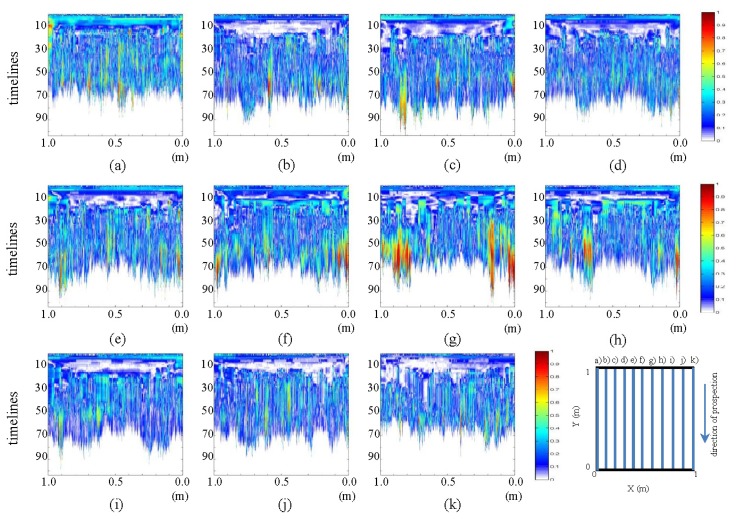
Vertical profiles: contrast between pre-processed images. (**a–k**) are profiles s12 to s22, respectively.

In this section, we present a number of contrasts between the pre-processed profiles for the initial and final states. In Figure 17 and 18, contrasts for the horizontal and vertical profiles, respectively, are presented.

In Figure 17, images (a) and (k), the mark that was observed in the interpretations already made can be easily seen. This mark corresponds to the introduction of water at WI and WO. Additionally, in the same pictures, an anomaly in the form of an ellipse at the bottom can be appreciated. This shape corresponds to the water in the pipe. Moreover, various profiles reveal the formation of the ellipse whose center is highly intense and which fades toward the ends. In these profiles, we see how the color intensity decreases as we near the central profile (leak location profile). Similarly, in images (e) and (g), one can observe the formation of a new ellipse that is located just above the ellipse corresponding to the water in the pipe. We also note that in image (f), this form is a hyperbola enveloping the ellipse corresponding to the area of water in the pipe. This form corresponds to leaked water, part of which has risen by capillarity to the surface of the ground.

In Figure 18, images (e), (f) and (g), one can observe the generation of an ellipsoidal shape that increases in size and consistency, as it travels from (e) to (g). Note that profile (g) is closest to the location of the leak.

## 7. 3D Comparison of the Analysis of Contrasts between Raw and Pre-Processed Images

**Figure 19.**
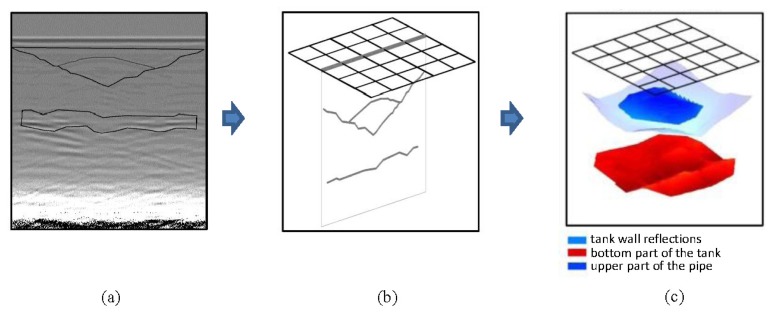
Example of 3D model construction: (**a**) GPR interpretation of raw data; (**b**) contour extraction; and (**c**) 3D model rendering.

In this section, we perform the extraction of the contours of the images resulting from the previously conducted contrasts. With these contours, two 3D models of contrasting performances in raw images and pre-processed images of the water in the pipe and leaked water are obtained. Contour extraction and the corresponding 3D representation have been developed in MatLab. In Figure 19, we present a simple scheme of one of the processes, whose two main steps are described as follows:

### GPR interpretations

Contours that can represent features of interest in the prospected underground are extracted either from the raw or the pre-processed images. When interpreting images of radargrams, the features most commonly analyzed are hyperbolas. They are demarcated by the color intensity within the image. A non-automatic process is proposed in this document with the aim of determining the feasibility of the interpretation of GPR data. In addition, we are interested in observing if these interpretations are reliable and can gain relevance for understanding features of leaks in WDSs. However, if this process is effective, it could be implemented automatically.

### Fusion process and 3D model

The fusion of the GPR image interpretations is achieved by their projection onto a common space. Firstly, a classification of common zones in the GPR interpretations that may correspond to specific sections of the area or volume is performed. Then, a mesh in the 3D space is constructed. Each of these volumes or surfaces is then added to a common space, shrinking, thus, into the 3D model. There are a number of applications to achieve this kind of fusion employed in photography or painting [19], which can be adapted to generate 3D models of the GPR interpretations.

**Figure 20.**
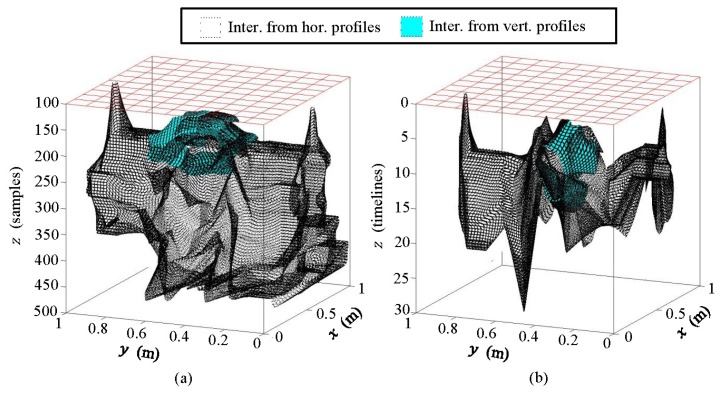
Comparison between 3D models generated from the interpretation of the horizontal and the vertical profiles contrasting the initial and the final states: (**a**) 3D model obtained from raw images; (**b**) 3D model obtained from pre-processed images.

In Figure 20, we present the 3D models.

The 3D models presented in Figure 20 show clear agreement with the schematic approach performed. It is also clear that the models are related, although their depth axis differs. Obviously, this is because they come from different images. In both cases, the water in WI and WO and the water in the pipe can be clearly observed. The leakage is also identified, but more consistently in (b) than in (a). It can be seen that 3D models produce more measurable results (numerically) and more comprehensible interpretation than flat images.

## 8. Analysis of Field Images: A Case Study

**Figure 21.**
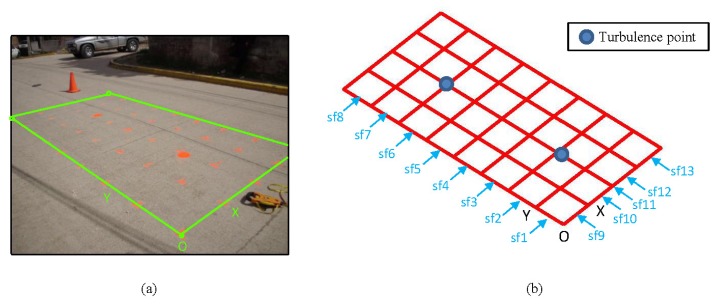
Layout for the fieldwork.

This section discusses the use of the multi-agent methodology, proposed in the previous section, in a real case. The fieldwork was conducted in an urban water supply system on a section of pipe, whose location was known. Using a geophone, turbulence points (where it was supposed that there was a water leak) were detected, and tests on the area near the pipe were performed using GPR. The roadway is hydraulic concrete and the pipe is 400-mm cast iron. A structure of paths similar to those in the laboratory tests was demarcated on the roadway (Figure 21a). The grid-mesh was 0.50 m. The area was 4.0 m by 2.0 m long in *x* and *y*, respectively. The profiles obtained with the survey were named sf1 to sf8 and sf9 to sf13 for horizontal and vertical lines, respectively (Figure 21b). It should be mentioned that the pipe is located in the sf11 profile. The GPR equipment used and the capture parameters correspond to the same features of the equipment used in the laboratory tests. However, in this case, the antenna used had a central frequency of 400 MHz.

**Figure 22.**
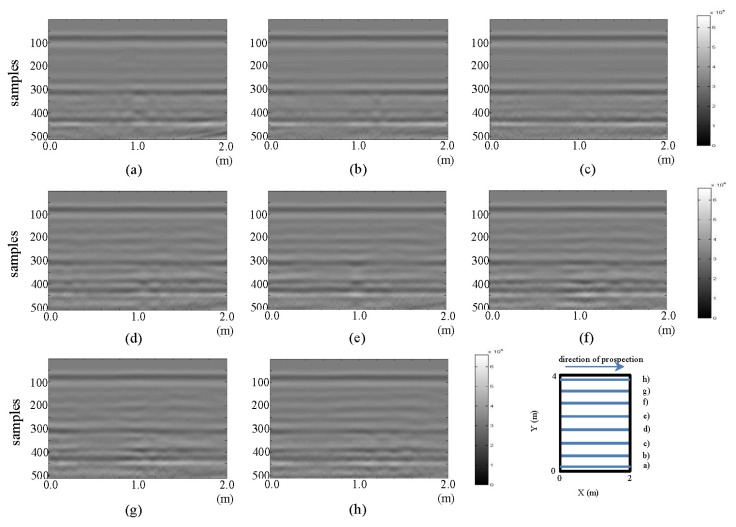
Horizontal profiles: raw images, (**a–h**) are profiles sf1 to sf8, respectively.

**Figure 23.**
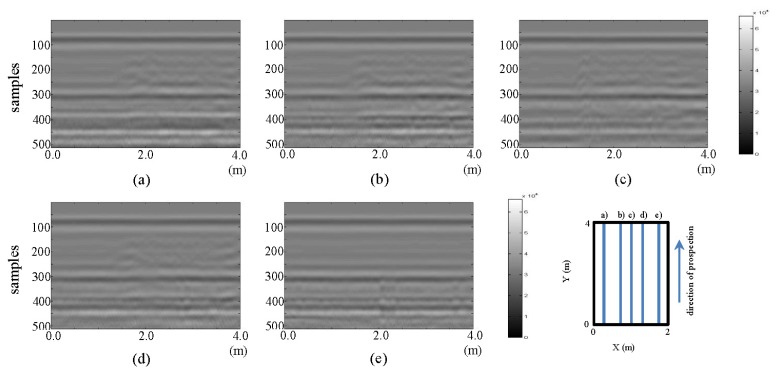
Vertical profiles: raw images. (**a–e**) are profiles sf9 to sf13, respectively.

In Figures 22 and 23, the raw images for the horizontal and vertical profiles, respectively, are presented.

In Figures 22 and 23, the observation of anomalies is deemed very complex. Only a small perturbation from (a) to (d) in Figure 23 can be observed.

**Figure 24.**
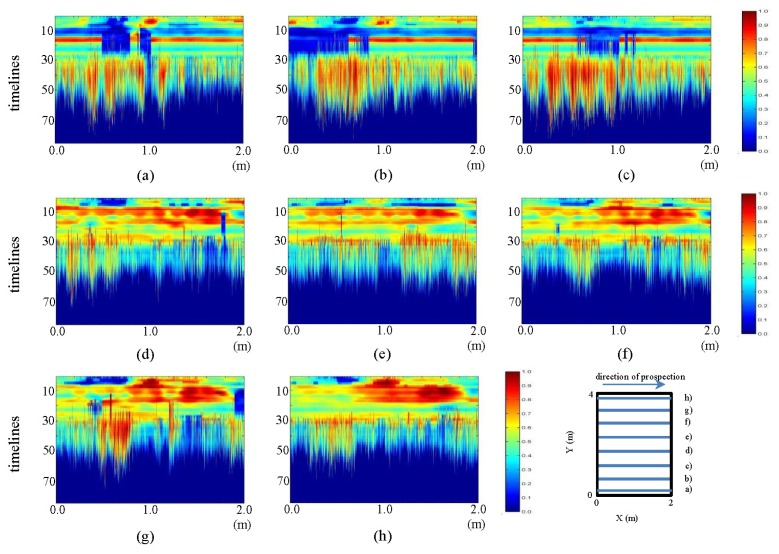
Horizontal profiles: pre-processed images. (**a–h**) are profiles sf1 a sf8, respectively.

**Figure 25.**
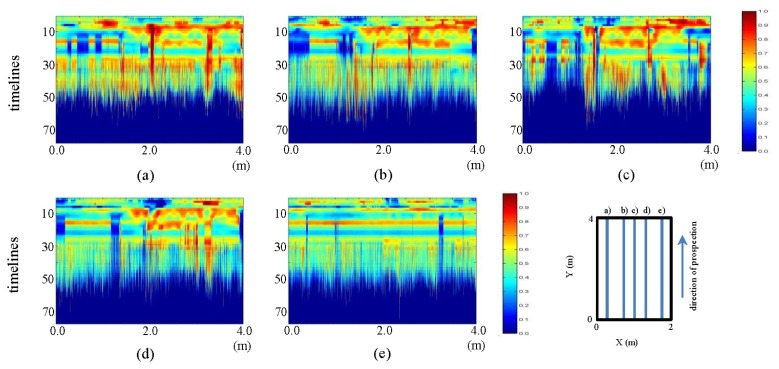
Vertical profiles: pre-processed images. (**a–h**) are profiles sf9 to sf13, respectively.

In Figures 24 and 25, we present the fieldwork preprocessed images corresponding to the horizontal and vertical profiles, respectively.

In Figures 24 and 25, the visualization improvement generated with the pre-processing can be clearly seen, making it possible to identify the elliptical shape of the pipe in the horizontal profiles (Figure 24), centered approximately on the position (1 m, five samples). Moreover, in this same figure, in pictures (d) to (h), a structure can be seen that starts at the bottom right of the ellipse corresponding to the pipe. This structure gains strength and is clearly demarcated as an ellipse in the direction (d) to (h). In Figure 25, two vertical structures at 1.5 m and 3.0 m, in sample 10, are also seen. These structures start from image (a), intensify towards image (c) and reduce their intensity in d), until finally disappearing in (e). This effect corresponds to the convolution observed in Figure 7.

**Figure 26.**
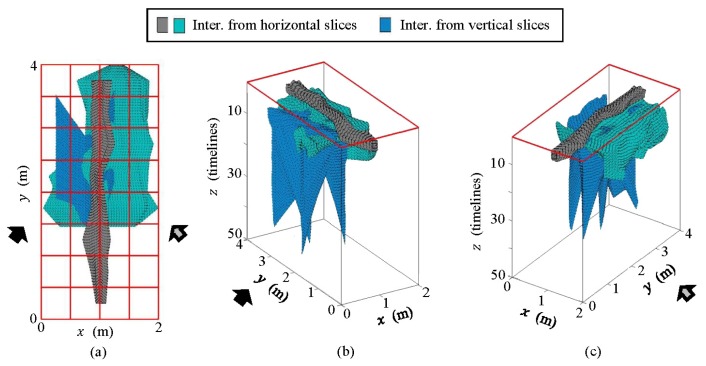
Interpretation from pre-processed images: 3D model, (**a**) azimuthal view; (**b**) lateral view #1; (**c**) lateral view #2.

Figure 26 shows the conjugation of these interpretations as 3D models, both for the pipe and likely leaks.

In Figure 26, water spots generated by the water leak in the pipe can be observed. These spots coincide with the turbulence points detected with the geophone.

## 9. Conclusions

By reducing the amount of water leaked, WDS managers can reduce the amount of money and energy wasted on producing or purchasing water. The use of various types of smart sensors to gather data and the application of advanced analytics, such as pattern detection, could provide valuable information on the location of leaks in the network. GPR, among other various sensors, can help detect potential leaks and abnormalities within WDSs.

In this work, two GPR tests have been conducted in laboratory conditions. One test employed a PVC pipe with no water inside and, obviously, without leaks, and the other test used the same pipe with water inside and some leakage. Additionally, we have conducted a field test in a leaking WDS. The different analyses show the difficulty of making interpretations from raw images. Indeed, it follows from these tests that introducing water into the system causes significant (numeric) differences that are not easily discernible from typical interpretations of the raw data obtained.

Moreover, this work shows that the application of appropriate preprocessing methodologies facilitates the visualization of features that are not reflected in the raw images. This is true both in the case of laboratory testing and in field cases. Consequently, we argue that the pre-processing used in this work facilitates the work of interpretation for non-highly qualified personnel in the use of GPR.

Additionally, it should be mentioned that in this type of pre-process, characteristics are obtained that are quantifiable (numerically). These characteristics can be the basis for further processing for the automatic classification of leaks in WDS.

Moreover, from these tests, we can conclude that the interpretations made using GPR are not merely subjective results. This is supported by the fact that there is consistency for all the results in 3D models between what is captured by GPR and what is expected. Thus, the reconstruction of 3D models from GPR analyses facilitates the interpretation of results.
